# Update on Recommendations for Use of Herpes Zoster Vaccine

**Published:** 2014-08-22

**Authors:** Craig M. Hales, Rafael Harpaz, Ismael Ortega-Sanchez, Stephanie R. Bialek

**Affiliations:** 1 Division of Viral Diseases, National Center for Immunization and Respiratory Diseases, CDC.

Herpes zoster vaccine (Zostavax [Merck & Co., Inc.]) was licensed in 2006 and recommended by the Advisory Committee on Immunization Practices (ACIP) in 2008 for prevention of herpes zoster (shingles) and its complications among adults aged ≥60 years (1). The Food and Drug Administration (FDA) approved the use of Zostavax in 2011 for adults aged 50 through 59 years based on a large study of safety and efficacy in this age group (2). ACIP initially considered the use of herpes zoster vaccine among adults aged 50 through 59 years in June 2011, but declined to recommend the vaccine in this age group, citing shortages of Zostavax and limited data on long-term protection afforded by herpes zoster vaccine (2). In October 2013, ACIP reviewed the epidemiology of herpes zoster and its complications, herpes zoster vaccine supply, short-term vaccine efficacy in adults aged 50 through 59 years, short- and long- term vaccine efficacy and effectiveness in adults aged ≥60 years, an updated cost-effectiveness analysis, and deliberations of the ACIP herpes zoster work group, all of which are summarized in this report. No vote was taken, and ACIP maintained its current recommendation that herpes zoster vaccine be routinely recommended for adults aged ≥60 years. Meeting minutes are available at http://www.cdc.gov/vaccines/acip/meetings/meetings-info.html.

*Recommendations for routine use of vaccines in children, adolescents, and adults are developed by the Advisory Committee on Immunization Practices (ACIP). ACIP is chartered as a federal advisory committee to provide expert external advice and guidance to the Director of the Centers for Disease Control and Prevention (CDC) on use of vaccines and related agents for the control of vaccine-preventable diseases in the civilian population of the United States. Recommendations for routine use of vaccines in children and adolescents are harmonized to the greatest extent possible with recommendations made by the American Academy of Pediatrics (AAP), the American Academy of Family Physicians (AAFP), and the American College of Obstetrics and Gynecology (ACOG). Recommendations for routine use of vaccines in adults are harmonized with recommendations of AAFP, ACOG, and the American College of Physicians (ACP). ACIP recommendations adopted by the CDC Director become agency guidelines on the date published in the* Morbidity and Mortality Weekly Report (MMWR). *Additional information regarding ACIP is available at http://www.cdc.gov/vaccines/acip.*

## Herpes Zoster Vaccine Background

The burden of herpes zoster increases as persons age, with steep increases occurring after age 50 years. Not only does the risk of herpes zoster itself increase with age, but among persons who experience herpes zoster, older persons are much more likely to experience postherpetic neuralgia (PHN) (3), non-pain complications (3), hospitalizations (4), and interference with activities of daily living (5). Because persons aged 50 years can expect to live an additional 32 years and persons aged 60 years, another 23 years (6), vaccination must offer durable effectiveness to protect against this increasing burden of disease.

Merck is the only U.S. supplier of varicella zoster virus (VZV)-containing vaccines (Zostavax; varicella vaccine [Varivax]; and combined measles, mumps, rubella, and varicella vaccine [MMR-V, ProQuad]). Beginning in 2007, Merck experienced production shortfalls of the bulk product used to manufacture VZV-based vaccines, leading to intermittent delays in filling of Zostavax orders. As a result of increased production capacity and reliability, by January 2012, Merck had resumed routine supply of varicella-containing vaccines, and Zostavax returned to normal shipping (7). As of August 2014, no subsequent supply disruptions have been reported.

## Studies of Efficacy and Duration of Protection

One randomized, placebo-controlled trial has evaluated short-term efficacy of herpes zoster vaccine administered to adults aged 50 through 59 years. This study of 22,439 adults in this age group showed a vaccine efficacy of 69.8% (95% confidence interval [CI] = 54.1%–80.6%) for the prevention of herpes zoster over a mean follow up period of 1.3 years (8). Efficacy for prevention of PHN and long-term vaccine efficacy in this age group were not studied.

Two studies have evaluated the short-term efficacy of the zoster vaccine in adults aged ≥60 years. The shingles prevention study (SPS) (9), a randomized controlled trial, followed 38,546 subjects for up to 4.9 years after vaccination (median = 3.1 years) and found a vaccine efficacy of 51.3% (CI = 44.2%–57.6%) for prevention of herpes zoster and 66.5% (CI = 47.5%–79.2%) for prevention of PHN. The short-term persistence substudy (STPS) (10) followed a subset of 14,270 SPS subjects primarily 4 to 7 years after vaccination and found a vaccine efficacy of 39.6% (CI = 18.2%–55.5%) for prevention of herpes zoster and 60.1% (CI = −9.8%–86.7%) for prevention of PHN. The point estimates for vaccine efficacy for prevention of herpes zoster by year after vaccination from the combined SPS and STPS studies decreased from 62.0% (CI = 49.6%–71.6%) in the first year after vaccination to 43.1% (CI = 5.1%–66.5%) in year 5. The 95% CIs around the point estimates for years 6 (30.6%) and 7 (52.8%) included zero; therefore vaccine protection could not be demonstrated after year 5. Vaccine efficacy for prevention of PHN decreased from 83.4% (CI = 56.7%–95.0%) in year 1 to 69.8 (CI = 27.3%–89.1%) in year 2. Estimates for years 3 through 7 after vaccination were not statistically significantly different from zero, although point estimates were generally higher compared with estimates of vaccine efficacy against herpes zoster.

The long-term persistence study (11) continued to follow 6,687 vaccinated subjects from STPS primarily from year 7 through year 10 after vaccination. By the end of the STPS, subjects in the placebo group had been vaccinated; therefore, no concurrent control group was available for comparison. Instead, a statistical model estimated herpes zoster and PHN incidence in a comparable unvaccinated group using historical SPS control subjects. The model estimated a vaccine effectiveness of 21.1% (CI = 10.9%–30.4%) for prevention of herpes zoster and 35.4% (CI = 8.8%–55.8%) for prevention of PHN over years 7 to 10 combined. Methodologic challenges in reliance on herpes zoster incidence in historical controls for calculation of vaccine effectiveness against herpes zoster include the fact that several studies (3,12–14) have shown increases in herpes zoster incidence over time. The lack of a concurrent control group seriously diminishes the strength of evidence for duration of vaccine protection from years 7 through 10. In addition, although some vaccine protection is demonstrated during the combined years 7–10 using this methodology, there is a high degree of uncertainty about trends in vaccine effectiveness over this time frame. For these reasons, effectiveness of herpes zoster vaccine administered to persons aged ≥60 years for preventing herpes zoster beyond 5 years remains uncertain.

## ACIP Review

At the October 2013 meeting, ACIP reviewed results from an updated cost-effectiveness analysis comparing health outcomes, health care resource utilization, costs, and quality-adjusted life years (QALYs) related to herpes zoster, PHN, and non-pain complications among unvaccinated persons and persons vaccinated at either age 50, 60, or 70 years (15). The model assumed waning of vaccine protection against herpes zoster to zero over 10 years for all ages, based on SPS, STPS, and long-term persistence study data. Projecting outcomes from ages 50 to 99 years, vaccination at age 60 years would prevent the most shingles cases (26,147 cases per 1 million persons) followed by vaccination at age 70 years and then age 50 years (preventing 21,269 and 19,795 cases, respectively). However, vaccination at age 70 years would prevent the most cases of PHN (6,439 cases per 1 million persons), followed by age 60 years and then age 50 years (preventing 2,698 and 941 PHN cases, respectively). From a societal perspective, vaccinating at age 70, 60, and 50 years would cost $37,000, $86,000, and $287,000 per QALY saved, respectively. The high cost per QALY saved with vaccination at age 50 years results from limited impact on prevention of PHN and other complications from ages 50 through 59 years and no remaining vaccine protection after age 60 when risk for PHN and other complications increases sharply. Results were robust in sensitivity analyses in which various more optimistic and pessimistic assumptions were made regarding waning of vaccine protection.

What recommendations are being reviewed?

Since 2008, the Advisory Committee on Immunization Practices (ACIP) has recommended routine vaccination of all persons aged ≥60 years with 1 dose of herpes zoster vaccine.

Why are the recommendations being reviewed now?

After approval by the Food and Drug Administration for use of zoster vaccine in adults aged 50 through 59 years in 2011, ACIP initially considered use of the vaccine among adults in this age group, but declined to change its recommendations at that time, citing shortages of Zostavax and limited data on long-term protection afforded by herpes zoster vaccine. A new review was conducted because the manufacturer has resumed routine supply of Zostavax and additional data on long-term protection have become available.

What is currently recommended?

Considering that the burden of herpes zoster and its complications increases with age and that the duration of vaccine protection in persons aged ≥60 years is uncertain, ACIP’s recommendation remains unchanged; herpes zoster vaccine is routinely recommended only for adults aged ≥60 years.

Because the protection offered by the herpes zoster vaccine wanes within the first 5 years after vaccination, and duration of protection beyond 5 years is uncertain, it is unknown to what extent persons vaccinated before age 60 years will be protected as they age and their risk for herpes zoster and its complications increases. Because duration of protection offered by the vaccine is uncertain, the need for revaccination is not clear. Assuming waning of vaccination protection according to currently available studies, the cost-effectiveness model projects a substantially greater reduction of disease burden, health care utilization, and costs with vaccination of older adults who have higher incidence of herpes zoster and related complications. Considering that the burden of herpes zoster and its complications increases with age and that the duration of vaccine protection in persons aged ≥60 years is uncertain, ACIP maintained its current recommendation that herpes zoster vaccine be routinely recommended for adults aged ≥60 years.

With FDA approval, Zostavax is available in the United States and indicated for use among adults aged ≥50 years. Vaccination providers considering the use of Zostavax among certain persons aged 50 through 59 years despite the absence of an ACIP recommendation should discuss the risks and benefits of vaccination with their patients. Although the vaccine has short-term efficacy, there have been no long-term studies of vaccine protection in this age group. In adults vaccinated at age ≥60 years, vaccine efficacy wanes within the first 5 years after vaccination, and protection beyond 5 years is uncertain; therefore, adults receiving the vaccine before age 60 years might not be protected when their risks for herpes zoster and its complications are highest. CDC is actively monitoring postmarketing data on duration of vaccine protection in adults vaccinated at age ≥60 years. As additional data become available, ACIP will reevaluate the optimal age for vaccination and the need for revaccination to maintain protection against herpes zoster and its complications.
